# Low continuation of antipsychotic therapy in Parkinson disease – intolerance, ineffectiveness, or inertia?

**DOI:** 10.1186/s12883-021-02265-x

**Published:** 2021-06-24

**Authors:** Thanh Phuong Pham Nguyen, Danielle S. Abraham, Dylan Thibault, Daniel Weintraub, Allison W. Willis

**Affiliations:** 1 Department of Neurology, University of Pennsylvania Perelman School of Medicine, 423 Guardian Drive, Blockley Hall 829, Philadelphia, PA 19104 USA; 2 Department of Neurology Translational Center for Excellence for Neuroepidemiology and Neurological Outcomes Research, University of Pennsylvania Perelman School of Medicine, Philadelphia, PA USA; 3 Center for Clinical Epidemiology and Biostatistics, University of Pennsylvania Perelman School of Medicine, Philadelphia, PA USA; 4 Center for Pharmacoepidemiology Research and Training, Center for Clinical Epidemiology and Biostatistics, University of Pennsylvania Perelman School of Medicine, Philadelphia, PA USA; 5 Department of Psychiatry, University of Pennsylvania Perelman School of Medicine, Philadelphia, PA USA; 6 Department of Biostatistics, Epidemiology, and Informatics, University of Pennsylvania Perelman School of Medicine, Philadelphia, PA USA

**Keywords:** Parkinson disease, Parkinson disease psychosis, Antipsychotics, Dopamine-receptor blockers, Pimavanserin, Medication continuation, Pharmacoepidemiology

## Abstract

**Background:**

Antipsychotics are used in Parkinson disease (PD) to treat psychosis, mood, and behavioral disturbances. Commonly used antipsychotics differ substantially in their potential to worsen motor symptoms through dopaminergic receptor blockade. Recent real-world data on the use and continuation of antipsychotic therapy in PD are lacking. The objectives of this study are to (1) examine the continuation of *overall* and *initial* antipsychotic therapy in individuals with PD and (2) determine whether continuation varies by drug dopamine receptor blocking activity.

**Methods:**

We conducted a retrospective cohort study using U.S. commercially insured individuals in Optum 2001–2019. Adults aged 40 years or older with PD initiating antipsychotic therapy, with continuous insurance coverage for at least 6 months following drug initiation, were included. Exposure to pimavanserin, quetiapine, clozapine, aripiprazole, risperidone, or olanzapine was identified based on pharmacy claims. Six-month continuation of *overall* and *initial* antipsychotic therapy was estimated by time to complete discontinuation or switching to a different antipsychotic. Cox proportional hazards models evaluated factors associated with discontinuation.

**Results:**

Overall, 38.6% of 3566 PD patients in our sample discontinued antipsychotic therapy after the first prescription, 61.4% continued with *overall* treatment within 6 months of initiation**.** Clozapine use was too rare to include in statistical analyses. *Overall* therapy discontinuation was more likely for those who initiated medications with known dopamine-receptor blocking activity (adjusted hazard ratios 1.76 [95% confidence interval 1.40–2.20] for quetiapine, 2.15 [1.61–2.86] for aripiprazole, 2.12 [1.66–2.72] for risperidone, and 2.07 [1.60–2.67] for olanzapine), compared with serotonin receptor-specific pimavanserin. *Initial* antipsychotic therapy discontinuation also associated with greater dopamine-receptor blocking activity medication use – adjusted hazard ratios 1.57 (1.28–1.94), 1.88 (1.43–2.46), 2.00 (1.59–2.52) and 2.03 (1.60–2.58) for quetiapine, aripiprazole, risperidone, and olanzapine, respectively, compared with pimavanserin. Similar results were observed in sensitivity analyses.

**Conclusions:**

Over one-third of individuals with PD discontinued antipsychotic therapy, especially if the initial drug has greater dopamine-receptor blocking activity. Understanding the drivers of antipsychotic discontinuation, including ineffectiveness, potentially inappropriate use, clinician inertia, patient adherence and adverse effects, is needed to inform clinical management of psychosis in PD and appropriate antipsychotic use in this population.

**Supplementary Information:**

The online version contains supplementary material available at 10.1186/s12883-021-02265-x.

## Background

Psychosis occurs in up to 60% of persons with Parkinson disease (PD), typically in later disease stages [1–3]. PD progression and treatment with dopaminergic medications are posited to drive PD psychosis (PDP) development; therefore, clinical guidelines recommend that outpatient PDP management begins with ruling out treatable or transient triggers such as infection or sleep disruption that can lead to reversible psychosis in older adults [4–6] and reducing or discontinuing anti-parkinsonian medications, if possible [7]. When additional measures are needed, antipsychotic (AP) therapy is recommended [7, 8]. Pimavanserin became the first AP approved by the United States (U.S.) Food and Drug Administration specifically and solely for PDP treatment in April 2016 [7, 8]. However, second-generation or atypical APs — clozapine, quetiapine, risperidone, olanzapine, and aripiprazole — have long been prescribed off-label for PDP [7–10], as well as for behavioral or mood disturbance in adults with neurodegenerative disease [11–13], although use for the latter indications and in older adult populations is discouraged by clinical safety guidelines [8, 14, 15].

Safety and tolerance data for AP use in PD patients has traditionally focused on dopamine receptor antagonism leading to worsening of parkinsonism. While the exact mechanism for the lower risk of parkinsonism among atypical APs is unknown, serotonin type 2A (5-HT2A) blockade, 5HT-2A: dopamine type 2 (D2) blockage ratio, and rate of association and dissociation at D2 receptors of atypical APs relative to typical APs are proposed explanations [16–20]. Aripiprazole, for example, is thought to carry a lower risk of extrapyramidal adverse effects due to its partial agonism at the D2 receptor in addition to 5HT-2A receptor antagonism [18, 19]. In contrast, pimavanserin is a selective 5-HT2A inverse agonist, with negligible binding at almost all other receptors targeted by atypical APs, and thus is expected to treat psychosis symptoms in PD without worsening motor symptoms [19, 21].

Data on the use and continuation of AP therapy in the PD population, which would be essential to understand clinical decision making and safety, is limited [22–24]. AP discontinuation or switching could be due to adverse effects, resolution of a provoked psychosis, or failure to respond to AP therapy [25, 26]. The objective of the current study is to determine (1) continuation of *overall* AP therapy (i.e., *any *AP medication regardless of initial medication prescribed) and (2) continuation of *initial* AP therapy (i.e., *initial* AP medication prescribed) and (3) patient, clinical and medication factors associated with AP discontinuation among PD patients.

## Methods

### Overview

We conducted a retrospective cohort study examining continuation of AP therapy in individuals with PDP, focusing on the most commonly prescribed atypical APs [3], using a large U.S. commercial health insurance database. The Office of Regulatory Affairs of the University of Pennsylvania (Philadelphia, Pennsylvania) granted Institutional Review Board exemption for this study. All methods were performed in accordance with the relevant guidelines and regulations.

### Data source

For this study, we used the 2001–2019 Optum’s de-identified Clinformatics® Data Mart Database [27]. Optum contains health care claims data from over 60 million commercially-insured persons across the U.S. [27] Available data include sociodemographic information (e.g., age, sex, race, income level, education level, etc.), medical encounters (e.g., inpatient or emergency department [ED] visits), and pharmacy prescription claims and laboratory results, among others [27]. Optum represents the commercially-insured U.S. population [28], allowing for a large-scale and diverse study of long-term medication use.

### Study population

#### Inclusion and exclusion criteria

We identified new users of pimavanserin, quetiapine, risperidone, aripiprazole, clozapine, or olanzapine from January 1, 2001, until June 30, 2019, using National Drug Codes from Multum Medisource Lexicon (Denver, Colorado). New users were defined as individuals without any prescription fills for *any* AP in the six-month baseline period before initiating one of the APs of interest [29, 30]. Individuals with loss of insurance coverage within 6 months of the first AP prescription were excluded. The first qualifying AP prescription was required to have a supply of at least 7 days, to minimize the potential for capturing planned short-term therapy.

Within this AP new user cohort, PD patients were identified as individuals (1) having at least two separate diagnosis claims for PD documented by International Classification of Diseases, 9th and 10th Revisions, Clinical Modification (ICD-9-CM and ICD-10-CM) diagnosis codes (i.e., 332, 332.0, and G20) [31, 32], and (2) with PD diagnosis preceding AP therapy. We restricted our sample to individuals aged 40–90 years. We excluded individuals with concurrent diagnosis codes for atypical parkinsonian syndromes (multiple systems atrophy, progressive supranuclear palsy, corticobasal degeneration), amyotrophic lateral sclerosis, dementia with Lewy bodies, schizophrenia, and bipolar disorder (Table S1) or, claims for long-term facility care within 6 months of starting AP therapy, because these groups would be expected to have differing clinical indications for and response to AP therapy, as well as variation in access to specialty care and management approach [33, 34] and our datasets do not have complete medication profile for individuals during their stay in long-term facilities.

#### Baseline covariates

Sociodemographic data, including age, sex, race/ethnicity (categorized as White, Black, Asian, Hispanic, and unknown or missing), and region of residence (i.e., Northeast, Midwest, South, West, or other) were captured at the time of the first AP prescription fill. Other covariates, measured using all data recorded in the 6 months before AP initiation, included the combined Charlson-Elixhauser comorbidity score [35, 36], the claims-based frailty index (CFI) [37], and the average numbers of (1) distinct medications filled per month, (2) outpatient visits with a neurologist, (3) ED visits, and (4) inpatient admissions.

#### Antipsychotic continuation

*Overall* continuation is defined as the continuation of AP therapy after the first AP prescription fill, regardless of the initial AP medication prescribed (i.e., patients are still considered as “continued AP therapy” if there is switching to another AP). To examine *overall* continuation of AP therapy, we identified all AP prescription fills occurring up to 6 months after AP initiation using pharmacy claims dates in Optum. Time to discontinuation of AP treatment was calculated from the initial AP prescription claim date until the end date for the last AP prescription using prescriptions' days supply, and allowing a standard 14-day grace period (i.e., prescription fill gap) between AP prescription fills [29, 38–40] to account for potential late refills. Individuals with no additional AP medication fill after the end of this grace period were categorized as discontinuing AP therapy.

We defined continuation of *initial* therapy as a continuation of the same AP medication initially prescribed. Similar principles were applied to examine continuation of *initial* AP therapy. Time to switch to new AP drug was calculated from the pharmacy claim date of the initial prescription through the expected end date of the last prescription fill of that same agent (plus the 14-day grace period). We only captured the first prescription fill for a different AP; examining multiple switches was beyond the scope of this study and not in alignment with our study objectives.

#### Categorizing antipsychotics by dopamine receptor antagonism

We categorized the atypical APs based on published estimates of D2 receptor occupancy for a given AP dose [20]. Per the literature, among atypical APs used in PDP, quetiapine has the lowest affinity for the D2 receptor, followed by clozapine, aripiprazole, and risperidone, with the highest D2 receptor antagonism measured in olanzapine [20]. We selected pimavanserin users as the reference group to facilitate our discussion regarding continuation of AP therapy, as this drug is thought to have no measurable dopaminergic receptor antagonism [19, 21].

### Statistical analyses

SAS v9.4 (Cary, North Carolina) was used to build the analytic dataset and analyze the study cohort. We defined an alpha level of 0.05 for all statistical tests. Baseline characteristics were compared between new initiators of pimavanserin, quetiapine, aripiprazole, risperidone, and olanzapine, using Chi-square or Kruskal-Wallis tests for categorical and continuous variables, respectively. We constructed Cox proportional hazards models to examine the association between patient, clinical and drug factors and the risk of discontinuation of *overall* and *initial* AP therapy. Kaplan-Meier curves were used to illustrate freedom from discontinuation of *overall* and *initial* AP therapy. To test the robustness of our initial findings, we performed several sets of sensitivity analyses in which we (1) extended the follow-up period to 12 months after AP initiation and (2) extended the grace period for prescription refills from 14 to 30 days, and (3) excluded all individuals with any ED or hospitalization within 6 months prior to initiation of AP therapy.

## Results

### Individual baseline characteristics

We identified 3566 individuals meeting our inclusion criteria:153 new users of pimavanserin, 2452 of quetiapine, 169 of aripiprazole, 462 of risperidone, and 304 of olanzapine. Clozapine use was extremely rare (only 26 users satisfying our inclusion and exclusion criteria) and thus was excluded from further statistical analyses. Table 1 displays demographic, clinical, and health care use characteristics of the final study sample. Individuals initiating aripiprazole were the youngest with a median age of 72 years (interquartile range [IQR] 64–78), whereas risperidone users were the oldest at 78 years (IQR 72–83) at initiation (*p* < 0.0001). Male users were the majority for all AP medications except aripiprazole (68.0% female) and risperidone (51.3% female). The majority of PD patients using APs were White (range: 60.0% [risperidone] – 65.7% [aripiprazole]), and the distributions of race category and geographical region were similar across individual AP drugs (*p* = 0.268, and *p* = 0.194, respectively).

**Table 1:** Characteristics of individuals with Parkinson disease initiated on antipsychotic therapy, Optum 2001–2019

Characteristic Type		Pimavanserin (***n*** = 153)	Quetiapine (***n*** = 2452)	Aripiprazole (***n*** = 169)	Risperidone (***n*** = 462)	Olanzapine (***n*** = 304)	***p-value***
Demographic	Age at initiation, years median (IQR)	75 (69–80)	77 (69–82)	72 (64–78)	78 (72–83)	76 (70–82)	**< 0.0001**^b^
	Age groups, n (col%)						**< 0.0001**^a^
	40–59 years	4 (2.6)	166 (6.8)	18 (10.7)	21 (4.5)	22 (7.2)	
	60 to 79 years	108 (70.6)	1389 (56.6)	119 (70.4)	249 (53.9)	179 (58.9)	
	≥80 years	41 (26.8)	897 (36.6)	32 (18.9)	192 (41.6)	103 (33.9)	
	Sex categories, n (col%)						**< 0.0001**^a^
	Male	94 (61.4)	1429 (58.3)	54 (32.0)	225 (48.7)	155 (51.0)	
	Female	59 (38.6)	1023 (41.7)	115 (68.0)	237 (51.3)	149 (49.0)	
	Race categories, n (col%)						0.268^a^
	White	96 (62.7)	1542 (62.9)	111 (65.7)	277 (60.0)	198 (65.1)	
	Nonwhite	31 (20.3)	450 (18.3)	27 (16.0)	100 (21.6)	41 (13.5)	
	Unknown/Missing	26 (17.0)	460 (18.8)	31 (18.3)	85 (18.4)	65 (21.4)	
	Region, n (col %)						0.194^a^
	Midwest	28 (18.3)	515 (21.0)	37 (21.9)	102 (22.1)	70 (23.0)	
	Northeast	12 (7.8)	287 (11.7)	22 (13.0)	63 (13.6)	31 (10.2)	
	South	70 (45.8)	984 (40.1)	81 (47.9)	183 (39.6)	115 (37.8)	
	West	42 (27.4)	657 (26.8)	29 (17.2)	114 (24.7)	88 (28.9)	
	Other	1 (0.6)	9 (0.4)	0 (0)	0 (0)	0 (0)	
Clinical	Combined Charlson-Elixhauser comorbidity score, mean (SD)	1.6 (1.9)	1.9 (2.4)	1.9 (2.1)	2.2 (2.5)	2.2 (2.4)	**0.0002**^b^
	Claims-based frailty index, mean (SD)	0.20 (0.05)	0.22 (0.06)	0.22 (0.06)	0.23 (0.07)	0.23 (0.07)	**< 0.0001**^b^
	CFI categories, n (col%)						**< 0.0001**^a^
	Robust	24 (15.7)	301 (12.3)	15 (8.9)	40 (8.6)	28 (9.2)	
	Prefrail	104 (68.0)	1526 (62.2)	100 (59.2)	253 (54.8)	173 (56.9)	
	Mildly frail	20 (13.1)	514 (21.0)	48 (28.4)	149 (32.3)	84 (27.6)	
	Moderate to severely frail	5 (3.3)	111 (4.5)	6 (3.5)	20 (4.3)	19 (6.3)	
Healthcare and medication use in 6 months prior to AP initiation	# Medications filled per month, mean (SD)	3.5 (1.8)	4.1 (2.4)	5.4 (2.7)	4.5 (2.5)	4.5 (2.5)	**< 0.0001**^b^
	# Neurology visits, mean (SD)	1.3 (1.2)	1.2 (1.6)	0.9 (1.2)	1.0 (2.7)	0.9 (1.3)	**< 0.0001**^b^
	# ED visits, mean (SD)	0.3 (0.7)	0.5 (1.4)	0.8 (2.5)	0.6 (1.2)	0.7 (2.2)	**0.0484**^b^
	# Inpatient admissions, mean (SD)	0.5 (3.3)	1.3 (4.0)	1.4 (3.6)	2.2 (6.9)	2.0 (5.1)	**< 0.0001**^b^

*AP* antipsychotic, *CFI* claims-based frailty index, *ED* emergency department, *IQR* interquartile range, *SD* standard deviation

^a^ Chi-square for categorical variables; ^b^ Kruskal-Wallis for continuous variables

The mean combined Charlson-Elixhauser comorbidity score differed significantly (*p* = 0.0002) across AP medications. However, the maximum difference of 0.6 points was well below the 2–3-point difference threshold associated with measurable variations in mortality and other health outcomes [35, 36]. Most study subjects met criteria for being “pre-frail” according to the CFI (54.8% [risperidone] – 68.0% [pimavanserin]). Quetiapine and pimavanserin users differed from initiators of the other APs by being more frequently categorized as “robust” (not frail) or “pre-frail” (*p* < 0.0001); having fewer ED visits and hospitalizations, more outpatient neurology visits, and fewer medications in the 6 months prior to AP initiation (*p*-values< 0.05).

### Continuation of *overall* antipsychotic therapy

Overall, 38.6% of 3566 individuals in our PD sample discontinued AP therapy after the first prescription; 61.4% continued with *overall* therapy within 6 months of initiation. As shown in Table 2, the six-month AP discontinuation varied by initial drug choice (*p* < 0.0001). *Overall* AP therapy discontinuation was lowest among pimavanserin initiators (22.9%), and highest among individuals initially treated with risperidone (42.2%). The median time to discontinuation of *overall* AP therapy was greatest among individuals started on pimavanserin at 168 days (IQR 71–213), followed by persons started on quetiapine (96.5 days, IQR 44–203), aripiprazole (85 days, IQR 44–190), and olanzapine (78 days, IQR 44–193.5). Risperidone therapy initiators had the shortest duration of therapy (72 days, IQR 44–186). Of note, 26 individuals starting on clozapine had the longest median duration to discontinuation of *overall* therapy at 200 days (IQR 61–209).

**Table 2:** Treatment status after first antipsychotic prescription fill and time to discontinuation of *overall* antipsychotic therapy

	Pimavanserin (***n*** = 153)	Quetiapine (***n*** = 2452)	Aripiprazole (***n*** = 169)	Risperidone (***n*** = 462)	Olanzapine (***n*** = 304)	***p-value***
***Treatment status after first AP prescription fill***						
Discontinued *overall* AP therapy, n (col%)	35 (22.9)	960 (39.2)	69 (40.8)	195 (42.2)	111 (36.5)	**0.0006**^a^
Continued *overall* AP therapy, n (col%)	118 (77.1)	1492 (60.8)	100 (59.2)	267 (57.8)	193 (63.5)	
Continued initial AP medication	105 (68.6)	1369 (55.8)	92 (54.4)	225 (48.7)	161 (53.0)	**0.0005**^a^
Switched to another AP medication	13 (8.5)	123 (5.0)	8 (4.7)	42 (9.1)	32 (10.5)	**< 0.0001**^a^
Median time to discontinuation of *overall* AP therapy, days (IQR)	168 (71–213)	96.5 (44–203)	85 (44–190)	72 (44–186)	78 (44–193.5)	**< 0.0001**^b^
Average supply of initial AP Rx, days (SD)	32.8 (12.6)	35.8 (19.8)	37.6 (22.2)	32.0 (15.5)	33.7 (19.4)	**0.0007**^b^

*AP* antipsychotic, *IQR* interquartile range, *Rx* prescription, *SD* standard deviation

^a^ Chi-square for categorical variables; ^b^ Kruskal-Wallis for continuous variables

**Fig. 1:**
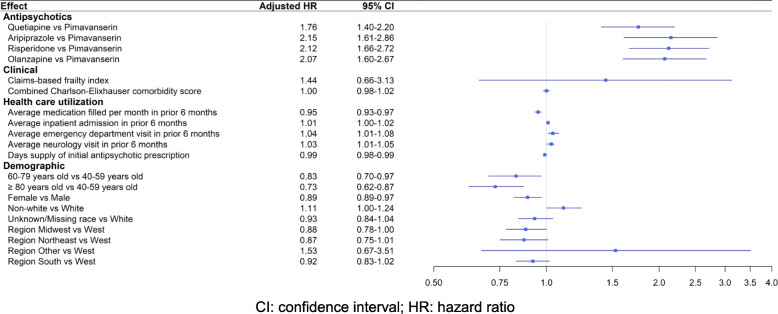
Factors associated with risk of *overall* antipsychotic discontinuation within 6 months among Parkinson disease patients

*Overall* AP discontinuation was less likely in higher age groups as compared to those aged < 60: adjusted hazard ratios (AHRs) 0.83 (95%CI 0.70–0.97) in the 60–79 years group and 0.73 (95%CI 0.62–0.87) in the ≥80 years group, as well as in females (AHR 0.89, 95%CI 0.89–0.97) as compared to males. Increasing frequency of ED or outpatient neurology visits was associated with a slightly higher risk of discontinuation (AHRs 1.04, 95%CI 1.01–1.08, and 1.03, 95%CI 1.01–1.05, respectively). After adjusting for study covariates, the estimated AHRs for *overall* AP discontinuation were 1.76 (95% CI 1.40–2.20) for quetiapine, 2.15 (95%CI 1.61–2.86) for aripiprazole, 2.12 (95%CI 1.66–2.72) for risperidone, 2.07 (95%CI 1.60–2.67) for olanzapine as compared to pimavanserin, as shown in Fig. 1. The Kaplan-Meier curve for the length of time after initiation of APs of interest until discontinuation of *overall* AP therapy also showed a statistically significant difference in the freedom from discontinuation times between the five groups (*p* < 0.0001) as shown in Figure S1.

### Continuation of *initial* antipsychotic therapy

Switching from one AP to another was much less common than stopping treatment altogether; around 6.0% of subjects switched. AP drug changes occurred most frequently among initiators of olanzapine (10.5%), and least frequently among those taking quetiapine first (4.9%). As shown in Table 3, persons prescribed pimavanserin, aripiprazole, risperidone, and olanzapine, who switched to another AP, overwhelmingly switched to quetiapine (91.7, 75.0, 66.7, and 62.5%, respectively). PD patients initially prescribed quetiapine who switched were most often given risperidone (28.3%), pimavanserin (26.7%), or olanzapine (20.0%). Of note, 5/26 (19.2%) of individuals initially started on clozapine switched to another AP within 6 months, mostly to pimavanserin (2 persons) or quetiapine (2 persons).

**Table 3:** Switching proportion and patterns from *initial* therapy within 6 months, stratified by *initial* antipsychotic prescribed

Initial Agent	Switched, n (row%)	Switching to drug, n (row %)					
		P	Q	A	R	O	Other antipsychotic
**Pimavanserin** (*n* = 153)	12 (7.8)	–	11 (91.7)	0 (0)	0 (0)	0 (0)	1 (8.3)
**Quetiapine** (*n* = 2452)	120 (4.9)	32 (26.7)	–	6 (5.0)	34 (28.3)	24 (20.0)	24 (20.0)
**Aripiprazole** (*n* = 169)	8 (4.7)	0 (0)	6 (75.0)	–	1 (12.5)	1 (12.5)	0 (0)
**Risperidone** (*n* = 462)	39 (8.4)	0 (0)	26 (66.7)	2 (5.1)	–	7 (17.9)	4 (10.3)
**Olanzapine** (*n* = 304)	32 (10.5)	2 (6.3)	20 (62.5)	1 (3.1)	6 (18.7)	–	3 (9.4)

*A* aripiprazole, *O* olanzapine, *P* pimavanserin, *Q* quetiapine, *R* risperidone

**Fig. 2:**
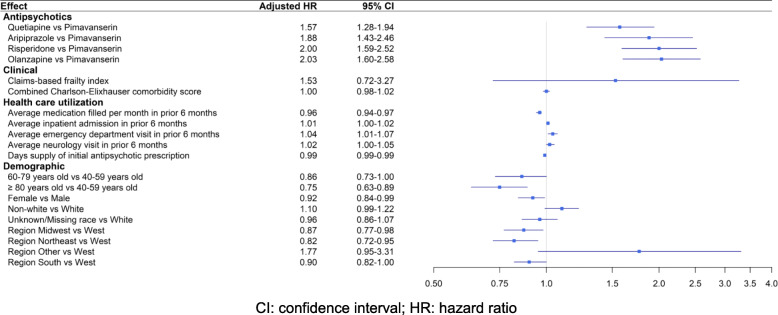
Factors associated with risk of *initial* antipsychotic discontinuation within 6 months among Parkinson disease patients

Discontinuation of *initial* AP was inversely associated with age ≥ 80 (AHR 0.75, 95%CI 0.63–0.89), but was not associated with race, sex, CFI, combined Charlson-Elixhauser comorbidity score, or incremental increases in recent average health care use. Both unadjusted and adjusted Cox proportional hazards models for the six-month follow-up and an allowable gap of 14 days between fills found a higher risk of discontinuation among PD patients initiated on quetiapine (hazard ratio [HR] 1.44, 95%CI 1.17–1.79; AHR 1.57, 95%CI 1.28–1.94), aripiprazole (HR 1.61, 95%CI 1.23–2.12; AHR 1.88, 95%CI 1.43–2.46), risperidone (HR 1.87, 95%CI 1.48–2.36; AHR 2.00, 95%CI 1.59–2.52), and olanzapine (HR 1.86, 95%CI 1.45–2.37; AHR 2.03, 95%CI 1.60–2.58) as compared to pimavanserin (Fig. 2). The Kaplan-Meier curve for the length of time after initiation of APs of interest until discontinuation of that specific AP also showed a statistically significant difference in the freedom from discontinuation times between the five treatment options (*p* < 0.0001) as shown in Figure S2.

Sensitivity analyses that expanded the observation window for discontinuation from 6 to12 months and the allowable refill gap from 14 to 30 days yielded similar findings as our primary analyses (Tables S2–S7). Individuals who had no ED visits or hospitalizations in the six months prior to AP therapy initiation also had similar discontinuation patterns.

## Discussion

Psychotic symptoms in PD are relatively common and consistently associated with negative health outcomes such as caregiver stress [41], nursing home placement [2, 42], and mortality [42]. Using real-world data, we examined the use of AP drugs most commonly prescribed to PD patients in the U.S. Our primary findings are (1) AP therapy is discontinued often and (2) AP therapy discontinuation and switching are greater among initiators of APs with complex receptor blocking properties, including greater dopamine-receptor blocking activity (i.e., quetiapine, aripiprazole, risperidone, and olanzapine), as compared to serotonin receptor-specific pimavanserin.

Our finding that almost 40% of individuals with PD stop *overall* AP treatment after the first prescription has multiple potential explanations. One potential explanation is an improvement in the symptoms for which the AP was prescribed. The first step in the detection and diagnosis of psychosis is to identify treatable or transient triggers. Infection, illness, sleep disruption, depression can lead to reversible psychosis in older adults [4–6]. Certainly, some of the PD patients in our sample could have had a psychosis cause identified and successfully addressed, with no need for long-term AP therapy. However, our sensitivity analyses suggested few patients in our sample had recent inpatient or emergent care that would reasonably be expected to be associated with clinical indications for temporary AP therapy. In contrast, psychosis due to PD-related processes may not respond to attempts to wean the AP prescribed [43].

Adverse drug events may also have played a role in the AP use patterns we observed. APs with the highest dopamine-receptor blocking potential (i.e., aripiprazole, risperidone, and olanzapine) had the lowest continuation of therapy and highest switching proportion as compared to pimavanserin and quetiapine; this finding could reflect drug intolerance or adverse effects [18, 19, 44]. Double-blind trials of olanzapine demonstrated no psychosis improvement and consistent worsening of motor function, even at low doses [8, 45]. A meta-analysis of risperidone treatment reported that one-third of PD patients experienced increased motor dysfunction [42, 46–48]. While aripiprazole is expected to have a lower risk of extrapyramidal adverse effects due to its high relative affinity for serotonergic receptors as compared to dopamine receptors [18, 19], emergent motor dysfunction in a single-arm open-label study of aripiprazole for PDP led to the study termination [7, 46, 49, 50]. Others who discontinued AP therapy may have done so because of a lack of response, partial response, or failure to meet patient/caregiver response expectations.

AP therapy use for behavioral symptoms and insomnia is strongly discouraged in geriatric clinical guidelines because it is likely to be ineffective and cause adverse effects [12, 14, 51–54]. Nevertheless, aborted trials of AP drugs for behavioral symptoms, such as agitation, pacing, yelling, sleep dysfunction, nocturnal restlessness, or insomnia, also likely account for a portion of the observed discontinuations. These symptoms are common in PD, especially in later disease stages, and cause significant caregiver distress [11, 55, 56]. Pimavanserin may be less likely to be used for behavioral management, as U.S. prescribers must provide medical documentation of psychosis as the reason for use [57]. If similar clinical documentation was required for all AP prescribing, the unmet need for management of behavioral and sleep disorders would become more evident, as would potentially inappropriate AP use. Future prospective studies will examine the frequency with which AP therapy is being used (in part or whole) for PD-related behavioral disturbances and measure clinical and safety outcomes associated with these off-label, potentially contraindicated uses.

### Study strengths

Our study had several strengths. Using a large healthcare database of commercially insured individuals in the U.S. [27] enabled us to examine AP prescribing in a diverse PD population sample, including older adults, women, and minorities, groups that are usually excluded from clinical trials. Prescription fills are well-captured in claims data and are preferred over self-reported medication use for adherence and persistence studies; the latter are subject to reporting and desirability bias [58]. Our methods for eligibility, exposure, and outcome measures are standard for pharmacoepidemiologic studies of treatment adherence, continuation, and persistence [29, 38–40]. Finally, our study provides comparative real-world data on all used medications, including the more recently approved pimavanserin; such multi-drug comparisons are only possible using real-world observational data.

### Study limitations

Despite these strengths, our study also has limitations. Optum contains commercially insured individuals, which often is associated with younger age, fewer or less severe comorbid conditions, and higher average income. Newer, more expensive, or more interaction-prone AP drug choices may therefore be over-represented in our sample. While Optum includes both commercially insured and Medicare Advantage individuals, our findings are expected to somewhat differ from a pure Medicare sample given differences in the formulary tier payment system, which can impact patient and physician’s decision to pursue a specific treatment. Moreover, we excluded patients with claims for long-term facility care within six months of starting AP therapy as we did not have access to their complete medication profile during their stay in these facilities, which should be further examined in another dataset. Additionally, Optum does not contain research instruments or clinical documentation (i.e., psychiatric evaluation results) of AP treatment response or the symptom profile. To address this limitation, it might be reasonable to consider large electronic health record (EHR) databases, although retrospective analyses of EHR data will still be subject to reporting bias [59]. Psychosis may improve with cholinesterase inhibitors (e.g., donepezil, rivastigmine, galantamine) [7], metacognitive therapy, or electroconvulsive therapy [60], but examining the use of these other treatments was beyond the scope of the current study. Furthermore, the relatively small sample size of pimavanserin compared to quetiapine, risperidone, or olanzapine prevented us from in-depth comparison between these groups. Finally, there was a potential for exposure misclassification due to the limited grace period allowed between prescription fills of APs.

## Conclusions

Our study highlights that continuation of AP therapy is generally low in PD patients. Future studies in other claims and clinical datasets are needed to confirm these findings and parse the contributing effects of treatment intolerance or ineffectiveness, prescribing appropriateness, patient non-adherence, and symptom resolution on psychosis treatment patterns, thus allowing for improved PDP management strategies.

## Supplementary Information

**Additional file 1: Figure S1.** Kaplan-Meier curve for freedom from discontinuation of overall antipsychotic therapy. **Figure S2.** Kaplan-Meier curve for freedom from discontinuation of initial antipsychotic therapy.

**Additional file 2: Table S1.** International Classification of Diseases, Ninth Revision, Clinical Modification (ICD-9-CM) and International Classification of Diseases, Tenth Revision, Clinical Modification (ICD-10-CM). **Table S2.** Cox proportional hazards model for overall antipsychotic discontinuation (6-month follow-up, 30-day grace period). **Table S3.** Cox proportional hazards model for overall antipsychotic discontinuation (12-month follow-up, 14-day grace period). **Table S4.** Cox proportional hazards model for overall antipsychotic discontinuation (12-month follow-up, 30-day grace period). **Table S5.** Cox proportional hazards model for initial antipsychotic discontinuation (6-month follow-up, 30-day grace period). **Table S6.** Cox proportional hazards model for initial antipsychotic discontinuation (12-month follow-up, 14-day grace period). **Table S7.** Cox proportional hazards model for initial antipsychotic discontinuation (12-month follow-up, 30-day grace period).

## Data Availability

De-identified Optum data used in this study are available to any interested researchers for purchase.

## Abbreviations

5-HT2A: serotonin type 2A
AHR: adjusted hazard ratio
AP: antipsychotic
CFI: claims-based frailty index
CI: confidence interval
D2: dopamine type 2
ED: emergency department
EHR: electronic health record
HR: hazard ratio
ICD-9-CM: International Classification of Diseases, 9th Revisions, Clinical Modification
ICD-10-CM: International Classification of Diseases, 10th Revisions, Clinical Modification
IQR: interquartile range
PD: Parkinson disease
PDP: Parkinson disease psychosis
U.S.: United States
