# Supplementary material for: Low continuation of antipsychotic therapy in Parkinson disease – intolerance, ineffectiveness, or inertia?
Source: BMC Neurol. 2021 Jun 24;21:240. doi: 10.1186/s12883-021-02265-x (PMC8223332; doi:10.1186/s12883-021-02265-x)
Supplement: Supplementary file 1 — Additional file 1: Figure S1. Kaplan-Meier curve for freedom from discontinuation of overall antipsychotic therapy. Figure S2. Kaplan-Meier curve for freedom from discontinuation of initial antipsychotic therapy. [file 12883_2021_2265_MOESM1_ESM.docx]

**Low Continuation of Antipsychotic Therapy in Parkinson Disease – Intolerance, Ineffectiveness, or Inertia?**

Thanh Phuong Pham Nguyen, PharmD, MBA, MSCE, Danielle S. Abraham, PhD, MPH, Dylan Thibault, MS, Daniel Weintraub, MD, Allison W. Willis, MD, MS

**SUPPLEMENTARY FIGURES**

**Figure S1.** Kaplan-Meier curve for freedom from discontinuation of *overall* antipsychotic therapy

**Figure S2.** Kaplan-Meier curve for freedom from discontinuation of *initial* antipsychotic therapy

**Figure S1. Kaplan-Meier curve for freedom from discontinuation of *overall* antipsychotic therapy**

**
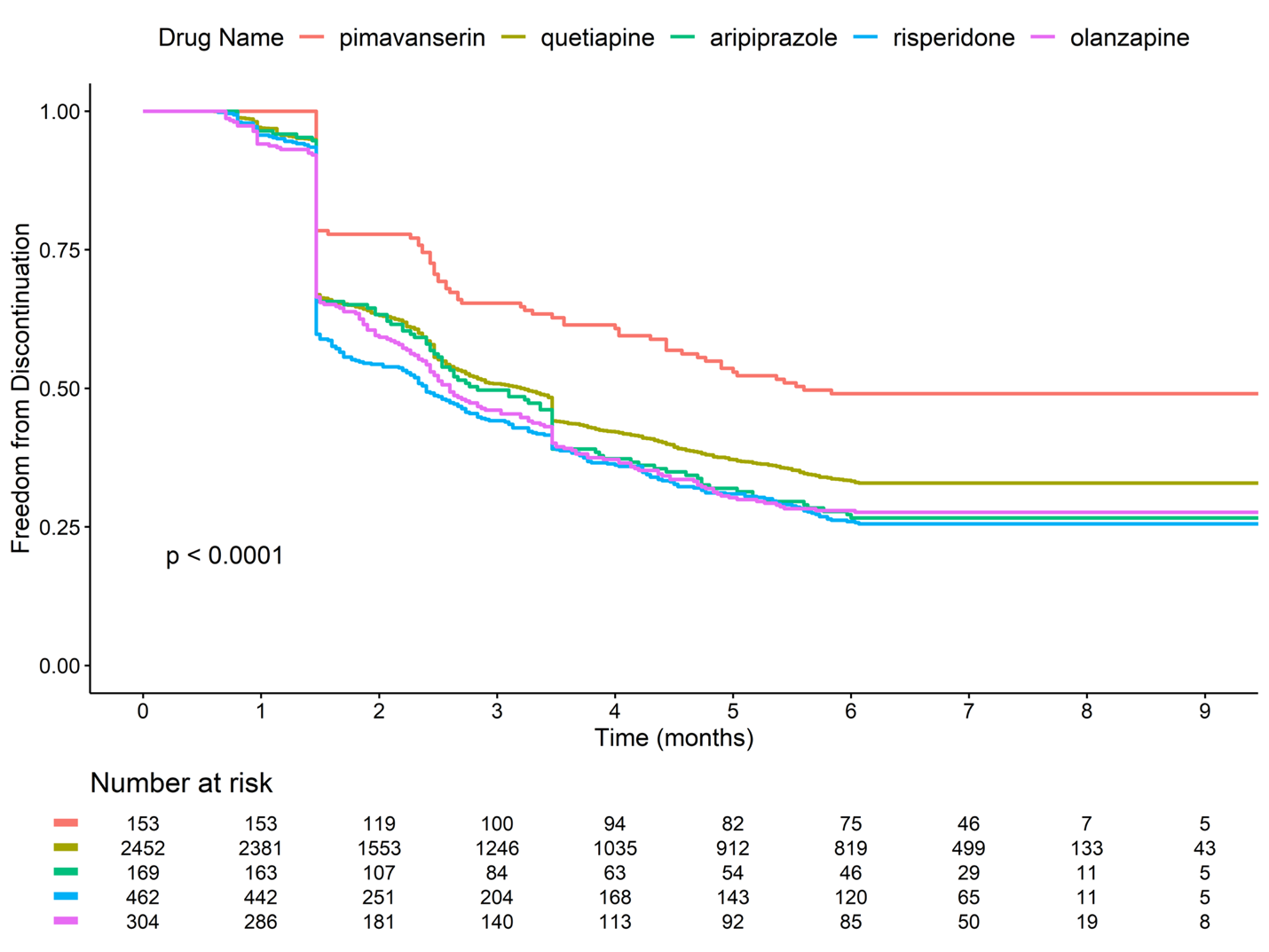
**

**Figure S2. Kaplan-Meier curve for freedom from discontinuation of *initial* antipsychotic therapy**

**
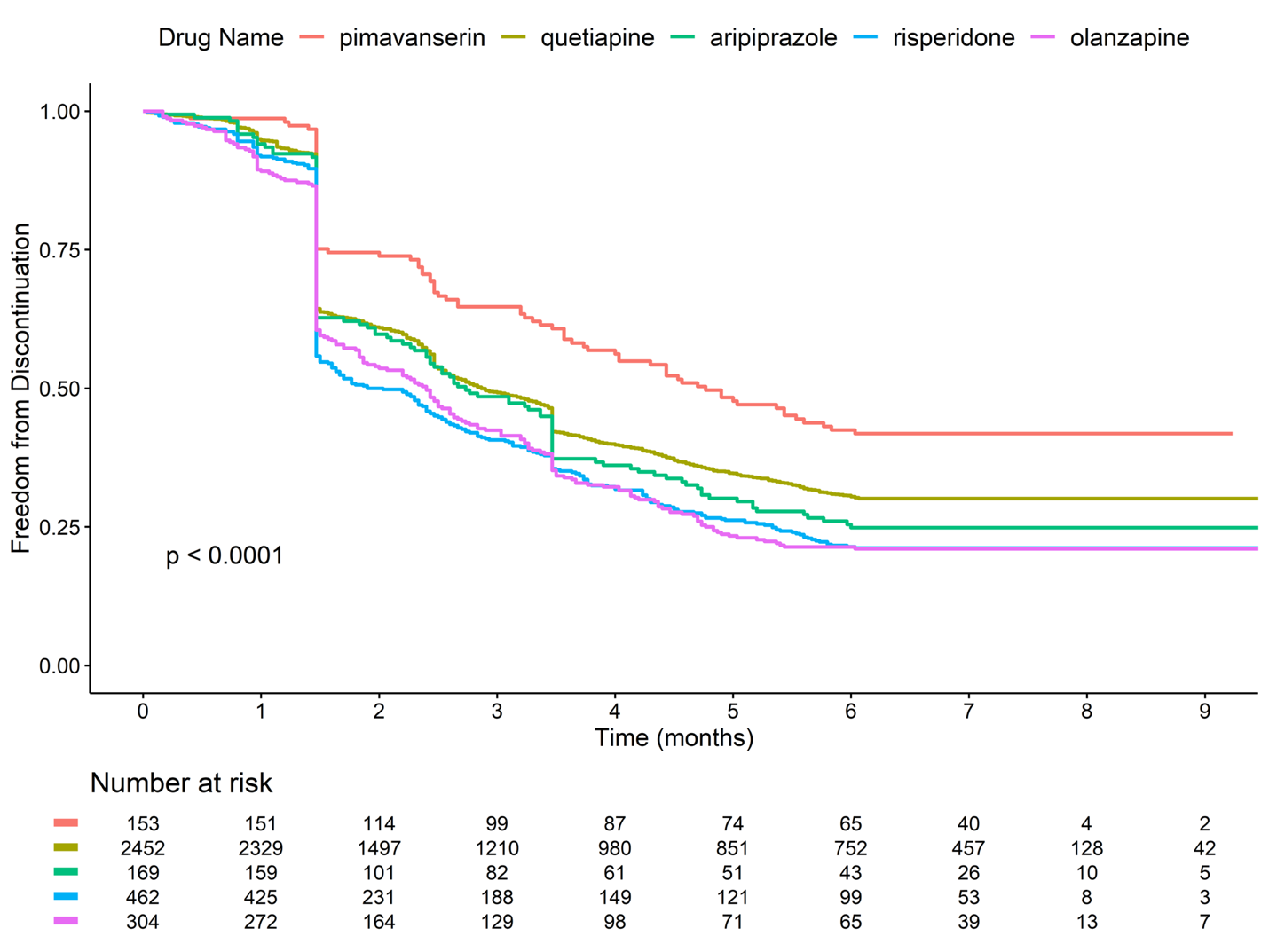
**
