# Supplementary material for: Low continuation of antipsychotic therapy in Parkinson disease – intolerance, ineffectiveness, or inertia?
Source: BMC Neurol. 2021 Jun 24;21:240. doi: 10.1186/s12883-021-02265-x (PMC8223332; doi:10.1186/s12883-021-02265-x)
Supplement: Supplementary file 2 — Additional file 2: Table S1. International Classification of Diseases, Ninth Revision, Clinical Modification (ICD-9-CM) and International Classification of Diseases, Tenth Revision, Clinical Modification (ICD-10-CM). Table S2. Cox proportional hazards model for overall antipsychotic discontinuation (6-month follow-up, 30-day grace period). Table S3. Cox proportional hazards model for overall antipsychotic discontinuation (12-month follow-up, 14-day grace period). Table S4. Cox proportional hazards model for overall antipsychotic discontinuation (12-month follow-up, 30-day grace period). Table S5. Cox proportional hazards model for initial antipsychotic discontinuation (6-month follow-up, 30-day grace period). Table S6. Cox proportional hazards model for initial antipsychotic discontinuation (12-month follow-up, 14-day grace period). Table S7. Cox proportional hazards model for initial antipsychotic discontinuation (12-month follow-up, 30-day grace period). [file 12883_2021_2265_MOESM2_ESM.docx]

**Low Continuation of Antipsychotic Therapy in Parkinson Disease – Intolerance, Ineffectiveness, or Inertia?**

Thanh Phuong Pham Nguyen, PharmD, MBA, MSCE, Danielle S. Abraham, PhD, MPH, Dylan Thibault, MS, Daniel Weintraub, MD, Allison W. Willis, MD, MS

**SUPPLEMENTARY TABLES**

# Table S1. International Classification of Diseases, Ninth Revision, Clinical Modification (ICD-9-CM) and International Classification of Diseases, Tenth Revision, Clinical Modification (ICD-10-CM)

# Table S2. Cox proportional hazards model for *overall* antipsychotic discontinuation (6-month follow-up, 30-day grace period)

# Table S3. Cox proportional hazards model for *overall* antipsychotic discontinuation (12-month follow-up, 14-day grace period)

# Table S4. Cox proportional hazards model for *overall* antipsychotic discontinuation (12-month follow-up, 30-day grace period)

# Table S5. Cox proportional hazards model for *initial* antipsychotic discontinuation (6-month follow-up, 30-day grace period)

# Table S6. Cox proportional hazards model for *initial* antipsychotic discontinuation (12-month follow-up, 14-day grace period)

# Table S7. Cox proportional hazards model for *initial* antipsychotic discontinuation (12-month follow-up, 30-day grace period)

# Table S1. International Classification of Diseases, Ninth Revision, Clinical Modification (ICD-9-CM) and International Classification of Diseases, Tenth Revision, Clinical Modification (ICD-10-CM) of excluded concurrent conditions

| Conditions | ICD-9-CM | ICD-10-CM |
| --- | --- | --- |
| Atypical parkinsonian syndrome | 3321, 3330, 3334, 09482 | G218, G2111, G2119, G230, G231, G232, G238, A5219 |
| Amyotrophic lateral sclerosis | 33520, 33521 | G1221 |
| Dementia with Lewy bodies | 33182 | G3183 |
| Schizophrenia | 29500, 29501, 29502, 29503, 29504, 29505, 29510, 29511, 29512, 29513, 29514, 29515, 29520, 29521, 29522, 29523, 29524, 29525, 29530, 29531, 29532, 29533, 29534, 29535, 29540, 29541, 29542, 29543, 29544, 29545, 29550, 29551, 29552, 29553, 29554, 29555, 29560, 29561, 29562, 29563, 29564, 29565, 29570, 29571, 29572, 29573, 29574, 29575, 29580, 29581, 29582, 29583, 29584, 29585, 29590, 29591, 29592, 29593, 29594, 29595 | F200, F201, F202, F203, F205, F2081, F2089, F209, F250, F251, F258, F259 |
| Bipolar disorder | 29600, 29601, 29602, 29603, 29604, 29605, 29606, 29610, 29611, 29612, 29613, 29614, 29615, 29616, 29640, 29641, 29642, 29643, 29644, 29645, 29646, 29650, 29651, 29652, 29653, 29654, 29655, 29656, 29660, 29661, 29662, 29663, 29664, 29665, 29666, 2967, 29680, 29681, 29682, 29689, 29690, 29699 | F3010, F3011, F3012, F3013, F302, F303, F304, F308, F309, F310, F3110, F3111, F3112, F3113, F312, F3130, F3131, F3132, F314, F315, F3160, F3161, F3162, F3163, F3164, F3170, F3171, F3172, F3173, F3174, F3175, F3176, F3177, F3178, F3181, F3189, F319, F338, F3481, F3489, F349, F39 |

**Table S2.** Cox proportional hazards model for *overall* antipsychotic discontinuation (6-month follow-up, 30-day grace period)

| **Factor** | **Unadjusted HRs (95% CI)** | **Adjusted HRs (95% CI)** |
| --- | --- | --- |
| **Pimavanserin** | REF | REF |
| Quetiapine | **1.84 (1.41-2.39)** | **2.02 (1.55-2.63)** |
| Aripiprazole | **1.83 (1.33-2.54)** | **2.25 (1.63-3.10)** |
| Risperidone | **2.15 (1.62-2.86)** | **2.34 (1.76-3.12)** |
| Olanzapine | **2.06 (1.53-2.78)** | **2.32 (1.73-3.12)** |
| **Age group** |  |  |
| 40 to 59 years |  | REF |
| 60 to 79 years |  | **0.78 (0.66-0.92)** |
| >=80 years |  | **0.69 (0.57-0.82)** |
| **Sex** |  |  |
| Male |  | REF |
| Female |  | **0.85 (0.78-0.93)** |
| **Race** |  |  |
| White |  | REF |
| Nonwhite |  | 1.11 (0.99-1.25) |
| Unknown/Missing |  | 0.94 (0.83-1.06) |
| **Region** |  |  |
| West |  | REF |
| Midwest |  | **0.84 (0.74-0.96)** |
| Northeast |  | **0.80 (0.68-0.94)** |
| South |  | **0.85 (0.76-0.95)** |
| Other |  | 1.40 (0.58-3.34) |
| Combined Charlson-Elixhauser comorbidity score |  | 1.01 (0.99-1.03) |
| Claims-based frailty index |  | 1.54 (0.66-3.60) |
| Average medications filled per month |  | **0.94 (0.92-0.96)** |
| Average neurology visits |  | **1.04 (1.02-1.06)** |
| Average emergency department visits |  | **1.04 (1.01-1.08)** |
| Average inpatient admission |  | 1.00 (0.99-1.01) |
| Days supply of initial antipsychotic fill |  | **0.99 (0.98-0.99)** |

**Table S3.** Cox proportional hazards model for *overall* antipsychotic discontinuation (12-month follow-up, 14-day grace period)

| **Factor** | **Unadjusted HRs (95% CI)** | **Adjusted HRs (95% CI)** |
| --- | --- | --- |
| **Pimavanserin** | REF | REF |
| Quetiapine | **1.32 (1.10-1.58)** | **1.41 (1.19-1.66)** |
| Aripiprazole | **1.49 (1.18-1.89)** | **1.73 (1.38-2.16)** |
| Risperidone | **1.66 (1.36-2.02)** | **1.76 (1.46-2.12)** |
| Olanzapine | **1.53 (1.23-1.89)** | **1.70 (1.39-2.07)** |
| **Age group** |  |  |
| 40 to 59 years |  | REF |
| 60 to 79 years |  | 0.84 (0.72-0.99) |
| >=80 years |  | 0.76 (0.65-0.90) |
| **Sex** |  |  |
| Male |  | REF |
| Female |  | **0.90 (0.84-0.97)** |
| **Race** |  |  |
| White |  | REF |
| Nonwhite |  | 1.09 (0.99-1.21) |
| Unknown/Missing |  | 0.96 (0.87-1.05) |
| **Region** |  |  |
| West |  | REF |
| Midwest |  | **0.88 (0.79-0.98)** |
| Northeast |  | 0.89 (0.78-1.01) |
| South |  | 0.92 (0.84-1.01) |
| Other |  | 1.19 (0.48-2.91) |
| Combined Charlson-Elixhauser comorbidity score |  | 0.99 (0.97-1.01) |
| Claims-based frailty index |  | 1.71 (0.86-3.39) |
| Average medications filled per month |  | **0.96 (0.94-0.97)** |
| Average neurology visits |  | 1.02 (1.00-1.04) |
| Average emergency department visits |  | **1.05 (1.03-1.08)** |
| Average inpatient admission |  | 1.01 (1.00-1.02) |
| Days supply of initial antipsychotic fill |  | **0.99 (0.99-0.99)** |

**Table S4.** Cox proportional hazards model for *overall* antipsychotic discontinuation (12-month follow-up, 30-day grace period)

| **Factor** | **Unadjusted HRs (95% CI)** | **Adjusted HRs (95% CI)** |
| --- | --- | --- |
| **Pimavanserin** | REF | REF |
| Quetiapine | **1.63 (1.33-2.01)** | **1.75 (1.43-2.14)** |
| Aripiprazole | **1.81 (1.40-2.35)** | **2.14 (1.67-2.74)** |
| Risperidone | **2.02 (1.61-2.52)** | **2.16 (1.73-2.69)** |
| Olanzapine | **1.84 (1.45-2.33)** | **2.02 (1.60-2.55)** |
| **Age group** |  |  |
| 40 to 59 years |  | REF |
| 60 to 79 years |  | **0.81 (0.69-0.95)** |
| >=80 years |  | **0.74 (0.63-0.88)** |
| **Sex** |  |  |
| Male |  | REF |
| Female |  | **0.87(0.80-0.94)** |
| **Race** |  |  |
| White |  | REF |
| Nonwhite |  | 1.08 (0.98-1.20) |
| Unknown/Missing |  | 1.00 (0.91-1.10) |
| **Region** |  |  |
| West |  | REF |
| Midwest |  | **0.82 (0.73-0.91)** |
| Northeast |  | **0.84 (0.73-0.95)** |
| South |  | **0.86 (0.78-0.95)** |
| Other |  | 1.27 (0.60-2.68) |
| Combined Charlson-Elixhauser comorbidity score |  | 1.01 (0.99-1.03) |
| Claims-based frailty index |  | 1.86 (0.91-3.81) |
| Average medications filled per month |  | **0.95 (0.94-0.97)** |
| Average neurology visits |  | **1.03 (1.01-1.06)** |
| Average emergency department visits |  | **1.05 (1.02-1.08)** |
| Average inpatient admission |  | 1.00 (1.00-1.01) |
| Days supply of initial antipsychotic fill |  | 0.99 (0.99-0.99) |

**Table S5.** Cox proportional hazard model for *initial* antipsychotic discontinuation (6-month follow-up, 30-day grace period)

| **Factor** | **Unadjusted HRs (95% CI)** | **Adjusted HRs (95% CI)** |
| --- | --- | --- |
| **Pimavanserin** | REF | REF |
| Quetiapine | **1.52 (1.20-1.93)** | **1.66 (1.31-2.10)** |
| Aripiprazole | **1.45 (1.07-1.96)** | **1.75 (1.30-2.35)** |
| Risperidone | **1.91 (1.47-2.46)** | **2.07 (1.60-2.67)** |
| Olanzapine | **1.99 (1.52-2.60)** | **2.18 (1.67-2.85)** |
| **Age group** |  |  |
| 40 to 59 years |  | REF |
| 60 to 79 years |  | **0.84 (0.72-0.99)** |
| >=80 years |  | **0.73 (0.62-0.87)** |
| **Sex** |  |  |
| Male |  | REF |
| Female |  | **0.88 (0.81-0.96)** |
| **Race** |  |  |
| White |  | REF |
| Nonwhite |  | 1.09 (0.97-1.22) |
| Unknown/Missing |  | 0.97 (0.87-1.09) |
| **Region** |  |  |
| West |  | REF |
| Midwest |  | **0.84 (0.74-0.96)** |
| Northeast |  | **0.76 (0.65-0.88)** |
| South |  | **0.84 (0.76-0.94)** |
| Other |  | 1.49 (0.72-3.09) |
| Combined Charlson-Elixhauser comorbidity score |  | 1.01 (0.99-1.04) |
| Claims-based frailty index |  | 1.59 (0.71-3.55) |
| Average medications filled per month |  | **0.95 (0.93-0.97)** |
| Average neurology visits |  | **1.04 (1.01-1.06)** |
| Average emergency department visits |  | 1.03 (1.00-1.07) |
| Average inpatient admission |  | 1.00 (0.99-1.01) |
| Days supply of initial antipsychotic fill |  | **0.99 (0.99-0.99)** |

**Table S6.** Cox proportional hazard model for *initial* antipsychotic discontinuation (12-month follow-up, 14-day grace period)

| **Factor** | **Unadjusted HRs (95% CI)** | **Adjusted HRs (95% CI)** |
| --- | --- | --- |
| **Pimavanserin** | REF | REF |
| Quetiapine | **1.23 (1.03-1.46)** | **1.32 (1.13-1.54)** |
| Aripiprazole | **1.38 (1.09-1.74)** | **1.59 (1.28-1.98)** |
| Risperidone | **1.63 (1.34-1.97)** | **1.73 (1.45-2.07)** |
| Olanzapine | **1.56 (1.27-1.92)** | **1.70 (1.40-2.06)** |
| **Age group** |  |  |
| 40 to 59 years |  | REF |
| 60 to 79 years |  | 0.91 (0.77-1.06) |
| >=80 years |  | **0.81 (0.69-0.95)** |
| **Sex** |  |  |
| Male |  | REF |
| Female |  | **0.91 (0.85-0.98)** |
| **Race** |  |  |
| White |  | REF |
| Nonwhite |  | 1.06 (0.96-1.17) |
| Unknown/Missing |  | 0.97 (0.88-1.06) |
| **Region** |  |  |
| West |  | REF |
| Midwest |  | **0.87 (0.78-0.96)** |
| Northeast |  | **0.84 (0.74-0.96)** |
| South |  | 0.92 (0.84-1.01) |
| Other |  | 1.52 (0.73-3.17) |
| Combined Charlson-Elixhauser comorbidity score |  | 1.00 (0.98-1.02) |
| Claims-based frailty index |  | 1.87 (0.95-3.70) |
| Average medications filled per month |  | **0.96 (0.95-0.98)** |
| Average neurology visits |  | 1.02 (1.00-1.04) |
| Average emergency department visits |  | **1.04 (1.01-1.07)** |
| Average inpatient admission |  | 1.01 (1.00-1.02) |
| Days supply of initial antipsychotic fill |  | **0.99 (0.99-0.99)** |

**Table S7.** Cox proportional hazard model for *initial* antipsychotic discontinuation (12-month follow-up, 30-day grace period)

| **Factor** | **Unadjusted HRs (95% CI)** | **Adjusted HRs (95% CI)** |
| --- | --- | --- |
| **Pimavanserin** | REF | REF |
| Quetiapine | **1.33 (1.10-1.61)** | **1.43 (1.20-1.70)** |
| Aripiprazole | **1.43 (1.12-1.83)** | **1.68 (1.34-2.11)** |
| Risperidone | **1.72 (1.39-2.12)** | **1.86 (1.53-2.26)** |
| Olanzapine | **1.66 (1.33-2.07)** | **1.81 (1.46-2.24)** |
| **Age group** |  |  |
| 40 to 59 yo |  | REF |
| 60 to 79 yo |  | 0.88 (0.75-1.03) |
| >=80 yo |  | **0.79 (0.67-0.93)** |
| **Sex** |  |  |
| Male |  | REF |
| Female |  | **0.89 (0.82-0.96)** |
| **Race** |  |  |
| White |  | REF |
| Nonwhite |  | 1.05 (0.95-1.16) |
| Unknown/Missing |  | 1.01 (0.92-1.11) |
| **Region** |  |  |
| West |  | REF |
| Midwest |  | **0.81 (0.72-0.90)** |
| Northeast |  | **0.81 (0.71-0.91)** |
| South |  | **0.86 (0.78-0.94)** |
| Other |  | 1.43 (0.77-2.68) |
| Combined Charlson-Elixhauser comorbidity score |  | 1.01 (0.99-1.03) |
| Claims-based frailty index |  | 1.87 (0.92-3.77) |
| Average medications filled per month |  | **0.96 (0.94-0.97)** |
| Average neurology visits |  | **1.03 (1.01-1.05)** |
| Average emergency department visits |  | 1.03 (1.00-1.06) |
| Average inpatient admission |  | 1.00 (0.99-1.01) |
| Days supply of initial antipsychotic fill |  | **0.99 (0.99-0.99)** |
